# The Highs and Lows of Memantine—An Autophagy and Mitophagy Inducing Agent That Protects Mitochondria

**DOI:** 10.3390/cells12131726

**Published:** 2023-06-27

**Authors:** Sholto de Wet, Asandile Mangali, Richard Batt, Jurgen Kriel, Nicola Vahrmeijer, Dana Niehaus, Rensu Theart, Ben Loos

**Affiliations:** 1 Department of Physiological Sciences, Stellenbosch University, Stellenbosch 7600, South Africa; 2 Department of Electric and Electronic Engineering, Stellenbosch University, Stellenbosch 7600, South Africa; 3 Microscopy Unit, Central Analytical Facility, Stellenbosch University, Stellenbosch 7600, South Africa; 4 Department of Psychiatry and Stikland Hospital, Faculty of Medicine and Health Sciences, Stellenbosch University, Cape Town 7530, South Africa

**Keywords:** mitochondrial function, autophagy, mitophagy, proteostasis, mitochondrial dynamics

## Abstract

Memantine is an FDA-approved, non-competitive NMDA-receptor antagonist that has been shown to have mitochondrial protective effects, improve cell viability and enhance clearance of Aβ_42_ peptide. Currently, there are uncertainties regarding the precise molecular targets as well as the most favourable treatment concentrations of memantine. Here, we made use of an imaging-based approach to investigate the concentration-dependent effects of memantine on mitochondrial fission and fusion dynamics, autophagy and mitochondrial quality control using a neuronal model of CCCP-induced mitochondrial injury so as to better unpack how memantine aids in promoting neuronal health. GT1-7 murine hypothalamic cells were cultured under standard conditions, treated with a relatively high and low concentration (100 µM and 50 µM) of memantine for 48 h. Images were acquired using a Zeiss 780 PS1 platform. Utilising the mitochondrial event localiser (MEL), we demonstrated clear concentration-dependent effects of memantine causing a protective response to mitochondrial injury. Both concentrations maintained the mitochondrial network volume whilst the low concentration caused an increase in mitochondrial number as well as increased fission and fusion events following CCCP-induced injury. Additionally, we made use of a customised Python-based image processing and analysis pipeline to quantitatively assess memantine-dependent changes in the autophagosomal and lysosomal compartments. Our results revealed that memantine elicits a differential, concentration-dependent effect on autophagy pathway intermediates. Intriguingly, low but not high concentrations of memantine lead to the induction of mitophagy. Taken together, our findings have shown that memantine is able to protect the mitochondrial network by preserving its volume upon mitochondrial injury with high concentrations of memantine inducing macroautophagy, whereas low concentrations lead to the induction of mitophagy.

## 1. Introduction

Neurodegenerative diseases are typically characterised by overlapping symptoms such as memory loss, mood changes and reduced muscle coordination. The brains of patients suffering from neurodegeneration are atrophied, and the neurons contain insoluble inclusion bodies. These inclusion bodies are composed of various constituents including autophagy receptors, ubiquitinated proteins and misfolded oligomeric proteins [1,2,3,4]. The wild-type forms of these misfolded proteins are key to normal neuronal functioning. However, misfolded proteins are aggregate-prone, and the resulting oligomers present in conformations are unable to pass through the proteasomal component of the UPS, making autophagy the primary means of protein degradation [5,6,7]. Additionally, the hydrophobic nature of oligomers enables their interaction with the membranes of multiple organelles, such as the endoplasmic reticulum and mitochondria, ultimately resulting in dysregulated calcium homeostasis as well as disrupted cellular bioenergetics [8,9]. Therefore, there is a need for autophagy flux, that is, the rate at which proteins and other cellular components are degraded through the autophagy pathway, to match the formation rate of these proteins to prevent proteotoxicity and the onset of cell death [10,11].

Mitochondria are key organelles responsible for cellular energy generation and regulate autophagy in accordance with the energetic demands of the cell [12,13]. This process is of particular importance in neurons due to their high ATP demands and protein synthesis and degradation requirements. The mitochondrial network is highly dynamic, and mitochondria have been shown to undergo fission and fusion events in response to metabolic stress [14,15,16]. Mitochondrial fusion events occur as a means of combining individual mitochondria and, in so doing, increase the ability for ATP generation, whereas fission events occur to segment regions of the mitochondria and are a means of isolating depolarised mitochondria for the purpose of their removal through mitophagy [17,18].

The accumulation of dysfunctional mitochondria is a hallmark of many neurodegenerative diseases, highlighting neuronal mitophagy dysfunction [19,20,21]. Similarly, the emerging role of mitochondrial uncoupling proteins and their role as a potential therapeutic target to preserve mitochondrial membrane potential and cellular energetic charge have received increasing attention [22]. Studies in which parkin, a regulatory protein critical for the initiation of mitophagy, has been overexpressed have shown the reestablishment of mitochondrial membrane potential and a reduction of dysfunctional mitochondria, possibly pointing towards a mitophagy receptor uncoupling [23]. Mitophagy is thus part of the basal mitochondrial quality control system and is of critical importance in the removal of dysfunctional mitochondria and the maintenance of mitochondrial network homeostasis.

It is known that Aβ_42_ interacts with the neuronal N-methyl-D-aspartate (NMDA) receptor, resulting in the excessive entry of calcium and causing excitotoxicity of the neuron [24,25]. Memantine, an FDA-approved drug typically prescribed for Alzheimer’s disease (AD) patients with mild to severe forms of AD, is a non-competitive inhibitor of the NMDA receptor which aids in the regulation of Ca^2+^ entry [26,27]. Additionally, memantine has been shown to induce autophagy, improve cell survival and decrease the generation of Aβ_42_ [28,29,30]. Although memantine elicits a concentration-dependent effect on cell survival, the degree of Aβ_42_ clearance, mitochondrial protection and optimal clinical application of this drug remains challenging. Memantine is registered for the treatment of moderate to severe AD and has shown a compound annual growth rate between 2008 and 2018 of 8.51% worldwide [31]. However, the risk–benefit relationship across the severity stages of AD remains poorly understood. The clinical use of lower or higher than recommended doses of memantine reflect the balancing act of neuronal survival-promoting pathways versus cell death-promoting pathways, the so-called NMDAR paradox [32]. To add to this complexity, a significant proportion of individuals are not being treated at the recommended daily dose for several possible reasons, including renal function adjustments, tolerability and variable adherence [33,34]. The question also arises whether sub-populations of patients with dementia might benefit from higher than recommended doses of memantine, especially taking into consideration the reported use of high doses (20–60 mg per day), albeit for shorter periods and in conditions other than dementia [35]. In light of these challenges, we sought to investigate the concentration-dependent effect of memantine in a neuronal cell model of CCCP-induced neurotoxicity [36]. Specifically, we made use of two concentrations of memantine that are considered relatively low and high in the in vitro context (50 µM and 100 µM) to investigate its potential concentration-dependent impact on the autophagy/lysosomal system and mitochondrial quality control [28]. Furthermore, we implemented a primarily quantitative microscopy-based approach where the role of memantine on mitochondrial abundance, volume and fission and fusion dynamics was assessed, taking advantage of a recently modified version of the MEL system [37]. Finally, autophagic flux was assessed, dissecting the autophagy pathway intermediates in terms of their respective cellular pool sizes, while the abundance of mitophagy events in each of the compartments, i.e., the autophagosome, autolysosome and lysosome, was dissected and confirmed using correlative light and electron microscopy (CLEM).

## 2. Methods and Materials

### 2.1. Cell Culture

GT1-7 cells were maintained in Dulbecco’s modified eagle’s medium (DMEM, ThermoFisher, Waltham, MA, USA, #41965062), supplemented with 10% foetal bovine serum (FBS, Sigma-Aldrich, #F0679) and 1% Penstrep (Sigma-Aldrich, St. Louis, MO, USA, #P4333) and were kept in a humidified incubator at 37 °C and 5% CO_2_. Cells were subcultured using trypsin (Sigma-Aldrich, #T4049) to detach cells from flasks. Afterwards, DMEM was added to the cells at a 2:1 ratio, and cells were collected into 15 mL Falcon tubes (Bio-Smart Scientific, Cape Town, South Africa #50050). Cells were centrifuged at 1500 rpm at room temperature for 3 min (Eppendorf, Hamburg, Germany, #5804R). Media was discarded and cells were resuspended in fresh DMEM. Cells were seeded either into T25 flasks (Bio-Smart Scientific, #70025), 8-chamber dishes (ThermoFisher, #Z734853) or onto gridded CLEM dishes (Mattek, MA, USA, P35G-1.5-14-C-GRD).

### 2.2. Treatment Interventions

All treatment interventions were made up using 1× PBS. Cells were incubated with memantine hydrochloride (Sigma-Aldrich, #PHR1886) for 48 h at working concentrations of 50 µM (LM) and 100 µM (HM), respectively, and memantine-treated media was refreshed every 24 h. To cause mitochondrial depolarisation and to assess potential rescue effects upon the mitochondrial network, carbonyl cyanide m-chlorophenylhydrazone (CCCP) (Sigma-Aldrich, #857815) was used at 10 µM for 6 h following the memantine treatment intervention. To assess for autophagy flux, cells were treated with 400 nM of bafilomycin A1 (Baf) (Sigma-Aldich, #B1793) for 4 h following memantine treatment intervention.

### 2.3. Fluorescence Microscopy and Image Acquisition for MEL

Cells were seeded onto 8-chamber dishes for image acquisition performed using a Carl Zeiss LSM780 ELYRA PS.1 super-resolution platform (Carl Zeiss, Oberkochen, Germany) using a LCI Plan Apochromat 100×/1.4 Oil DIC M27 objective. Cells were incubated in a master mix of pre-warmed media containing 1:1000 TMRE (ThermoFisher, #T669) and 1:200 Hoechst (Sigma-Aldrich, H6024) for 10 min prior to imaging. Images were acquired using 405 nm and 561 nm lasers, and raw images were captured as z-stacks containing 10 image micrographs with a z-step width of 0.25 µm taken over 9 time frames, acquired sequentially, automatically initiated immediately following completion of the previous z-stack acquisition. In total, no more than 30 s were required to complete a round of acquisition of each z-stack. Images were processed through a Fiji ImageJ 1.53 t pipeline that conducted deconvolution followed by contrast adjustment to ensure optimal thresholding followed by the thresholding execution. Binarized images were then processed and analysed using the mitochondrial event localiser (MEL) ImageJ plugin to detect and localise fission and fusion events [37], while also producing mitochondrial structure count and average structure volume (https://github.com/rensutheart/MEL-Fiji-Plugin (accessed on 1 February 2022)). To obtain the results presented, we utilised a total sample size of *n* = 9 cells, with each cell observed across 9 consecutive time points. The averaging of these time frames was performed independently for each cell, and the resulting averages were then further averaged across the 9 cells. This approach allowed us to capture the overall trends in mitochondrial dynamics within the treatment groups across three independent experiments.

### 2.4. Fluorescence Microscopy and Image Acquisition for Autophagy and Mitophagy Assessment

Cells were transfected with 5 µg GFP-LC3 plasmid (Addgene, Watertown, MA, USA #21073) using the NEON Electroporation System (Invitrogen, MPK5000) and NEON Transfection tool kit (Invitrogen, Waltham, MA, USA, MPK10096), according to the manufacturer’s instructions. Next, cells were seeded into 8-chamber dishes and left to adhere for 24 h before beginning the treatment protocol. Image acquisition was performed using a Carl Zeiss LSM780 ELYRA PS.1 super-resolution platform using a LCI Plan Apochromat 100×/1.4 Oil DIC M27 objective. Cells were then incubated in a master mix solution containing 75 nM LysoTracker Red (ThermoFisher, #L7528) and 75 nM MitoTracker DeepRed (ThermoFisher, #M22426) for 20 min prior to imaging. Images were acquired using 488 nm, 561 nm and 633 nm lasers, and lasers 561 and 633 were kept on separate tracks to avoid cross-excitation. Raw images were captured as z-stacks at 100× magnification using a minimum of 12 image frames with a step width of 0.35 µm. Acquired images were processed using an ImageJ macros pipeline to conduct deconvolution and image contrast adjustment before being processed through a Python pipeline that resulted in the thresholding of images and producing mitochondrial, lysosomal and autophagosomal counts and volume assessments as well as colocalised structure counts. This pipeline was a modified version of a previously utilised pipeline. During the image analysis, a size filter was applied to counteract background noise caused by the GFP-LC3 probe as well as to improve accuracy in vesicle colocalisation [38]. A total samples size of *n* = 9 cells was acquired in three independent experiments per treatment group.

We utilised regression-adjusted colocalisation colour mapping (RACC) [39] to precisely, qualitatively assess the extent and intracellular regions that showed a greater degree of colocalisation between the fluorescence intensities of mitochondria and lysosomes. Brighter regions in the visualisation correspond with regions where the fluorescence intensities are most similar, typically indicating stronger correlation and hence, a higher probability of true colocalisation, while darker regions manifest where the fluorescence intensities are divergent, indicating a lower probability of true colocalisation. Additionally, regions of colocalisation for which the underlying fluorescence intensities are lower will also appear darker relative to regions where the underlying intensities are higher. RACC requires a penalisation factor (θ) to be chosen, which determines how much intensity pairs that are far away from the regression line on the scatter plot should be attenuated in the final visualisation. We found that θ = 45° produced favourable results for our data, and this value was therefore applied to all our treatment groups to ensure most standardised comparison.

### 2.5. Correlative Light and Electron Microscopy (CLEM)

CLEM gridded coverslip dishes were treated with 10% collagen solution (Sigma-Aldrich, C8919) made in 1× PBS and left to dry overnight at 4 °C. Cells were then seeded and allowed to adhere overnight. Next, cells were treated, stained and subsequently fixed with 4% PFA for 10 min before being rinsed 3 times with sterile PBS and imaged using a Zeiss 780 confocal microscope. Tile scans were performed at 10× magnification to better locate areas of interest that were associated with respective grid numbers on the dish. The positions of each cell on the grid region of choice were then set and annotated to ensure accurate cut-outs of the resin block later [40]. Once a region of interest was identified, z-stacks of 30 slices were acquired of individual cells at a 100× magnification under 488 nm, 561 nm and 633 nm excitation, as well as a TPMT, to aid in subsequent CLEM overlay accuracy. Cells were fixed for electron microscopy and prepped according to [41], but in brief, cells were treated for 30 min at room temperature with a cocktail of 2.5% glutaraldehyde and 4% formaldehyde in 0.1 M Sorenson’s phosphate buffer. Thereafter, cells were incubated with 2% reduced osmium tetroxide (mixture of 4% OsO_4_ and 3% potassium ferricyanide, 1:1). Cells were further incubated with 0.03 M lead aspartate for 30 min at room temperature before dehydration in an ethanol series of increasing concentration for 5 min each on ice (20%, 50%, 70%, 90%, 100%, anhydrous 100%). The cells were then incubated twice with 100% Epon for 90 min at room temperature and prepared for ultramicrotoming using a Leica ultramicrotome (Leica Microsystems, Wetzlar, Germany) and Ultra 45° 3 mm diamond knife (Diatome US, Hatfield, PA, USA, MS16427). Ultrathin sections of 100 nm were cut and collected onto 5 × 5 mm silicon wafer squares (Agar Scientific Ltd., Essex, UK, G3390). Samples were acquired on the ThermoFisher Apreo Volumescope FESEM at 5 kV accelerating voltage and 1.6 nA probe current using the T1 trinity detectors with immersion lens use-case. Electron images were captured in TIF format at store resolution of 3072 × 2304 pixels. Once both fluorescence and electron image data sets were acquired, overlays were performed using the ec-CLEM plugin available in the Icy Bioimaging software (http://icy.bioimageanalysis.org/plugin/ec-CLEM (accessed on 1 August 2022) [42].

### 2.6. Statistical Analysis

Results were shown as mean values ± SEM, and statistical analysis was performed using GraphPad Prism v9.4.1. Whilst fission and fusion dynamics were analysed using a two-way ANOVA, all other results were analysed using a one-way ANOVA, both of which were followed by the Fisher’s LSD post-hoc test to assess for significance. Results were considered significant when observing a *p*-value less than 0.05.

## 3. Results

### 3.1. Evaluating the Fission and Fusion Events of the Mitochondrial Network

**Figure 1:**
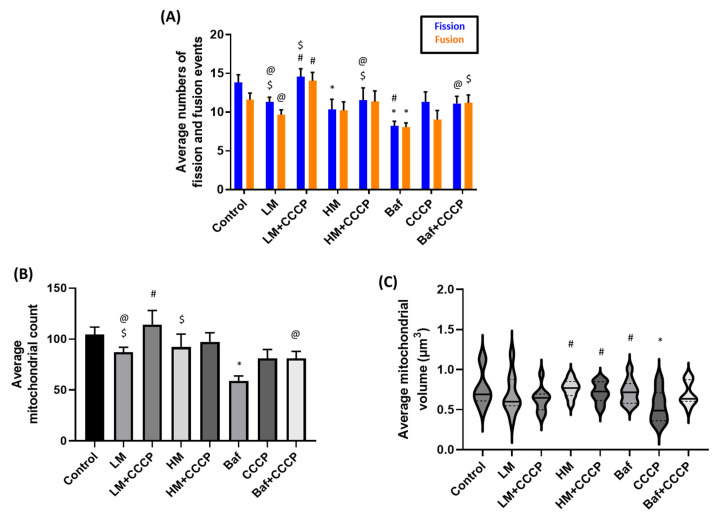

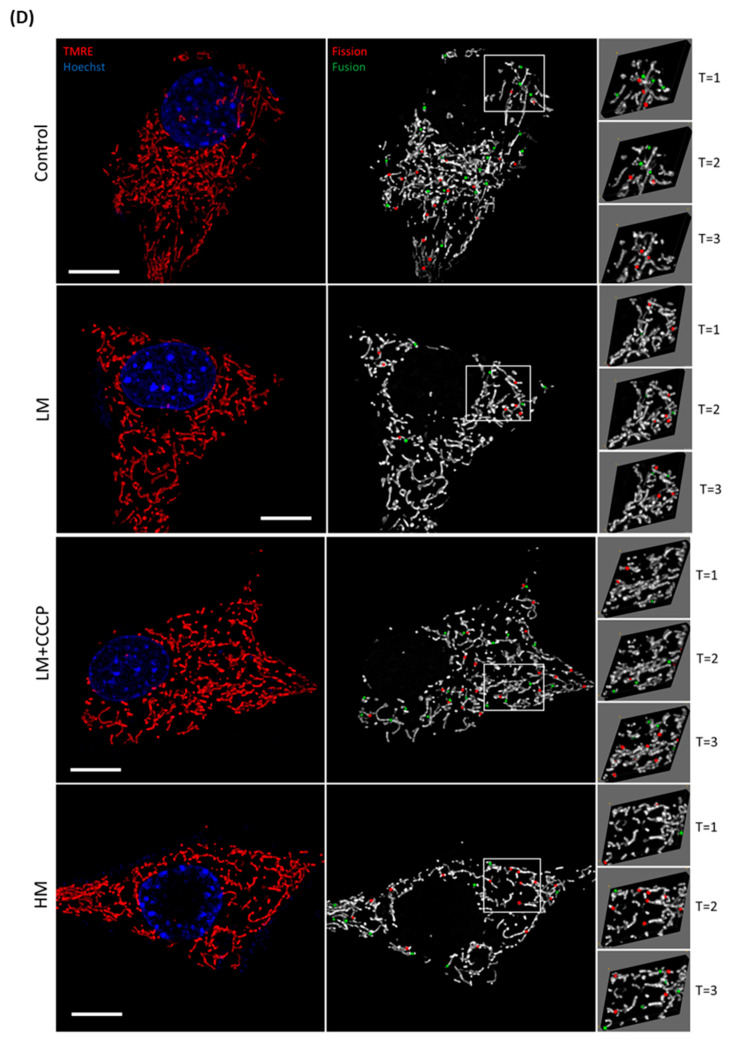

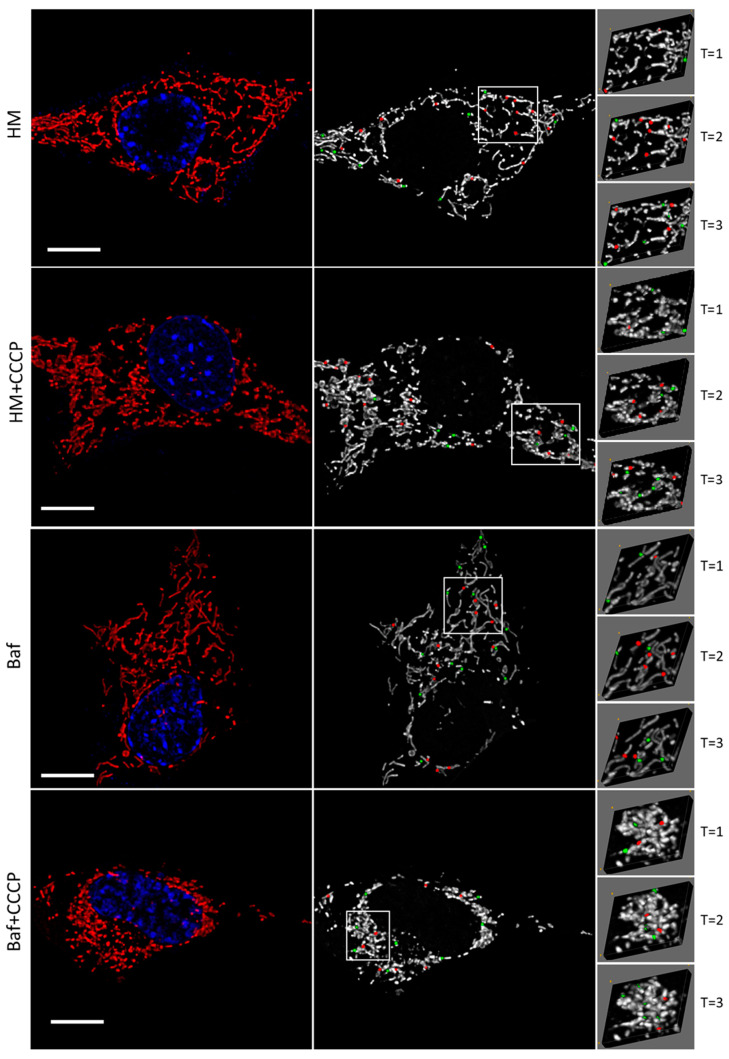
Memantine causes concentration-dependent changes to the mitochondrial network. Cells were stained and imaged using TMRE and Hoechst and then analysed using MEL to assess mitochondrial morphology and its response that occurred over time and in 3D. After treatment, the average number of fission (left bar) and fusion (right bar) events were measured (**A**). Subsequently, MEL was further used to assess the average mitochondrial structure counts (**B**) as well as the average volume changes (**C**). Representative MIP confocal micrographs are shown as well as the binarized mitochondrial image used to assess mitochondrial dynamics. Binarized micrographs bear the fission and fusion events, coloured puncta. Additionally, representative regions were selected and expanded upon to represent the 3D changes that occur in 3 subsequent time points assessed by MEL (**D**). The results depicted in the figure represent the average of 9 cells, where each cell was analysed across 9 consecutive time frames. The total number of cells analysed per treatment group was *n* = 9. Results are expressed as the mean ± SEM. Scale bar = 10 µm. * *p* < 0.05 vs. Control, # *p* < 0.05 vs. CCCP, $ *p* < 0.05 vs. Baf and @ *p* < 0.05 vs. LM + CCCP.

To understand the effects of memantine treatment on the mitochondrial network, we made use of the mitochondrial event localiser (MEL) plugin, allowing the quantitative assessment of mitochondrial fission and fusion dynamics [37]. This allowed us to observe the trends that occur for the cell to reach equilibrium and to better understand the dynamic changes that happen in a given cell. We analysed the average changes that occur in 9 cells per treatment group with each cell being measured over 9 consecutive time frames. Carbonyl cyanide m-chlorophenylhydrazone (CCCP) was used to disrupt the transmembrane potential and thereby depolarise the mitochondria, providing mechanistic insight into how memantine at either concentration may offer protection to the mitochondrial network. Bafilomycin (Baf) in combination with CCCP (Baf + CCCP) was used to assess the impact of mitophagy on the dynamics of the mitochondrial network [43]. LM + CCCP, but not HM + CCCP, exposure led to a significantly higher number of fission and fusion events compared to CCCP treatment only. Fission events of LM [11.3 ± 0.6 (*p* < 0.05)], LM + CCCP [14.6 ± 1.0 (*p* < 0.05)] and HM + CCCP [11.5 ± 1.6 (*p* < 0.05)] were significantly higher than Baf treated cells, whereas fusion events were significantly higher in CCCP + Baf [11.2 ± 1.0 (*p* < 0.05)] treated cells. CCCP treatment did not lead to a significant change in the fission or fusion events compared to the control (Con) (Figure 1A); however, Baf treatment caused a significant decrease in both fission [8.2 ± 0.6] and fusion events [8.1 ± 0.6 (*p* < 0.05)] compared to Con [13.9 ± 1.0 and 11.6 ± 0.9 (*p* < 0.05)] as well as well as exposure to LM + CCCP. No significant fission or fusion changes occurred following treatment with either 50 µM (LM) [11.3 ± 0.6 and 9.7 ± 0.6 (*p* < 0.05)] or 100 µM (HM) [10.4 ± 1.3 and 10.2 ± 1.1 (*p* < 0.05)] of memantine compared to Con.

### 3.2. Mitochondrial Structure Counts and Volume Assessment

Fission and fusion events are indeed dynamic and therefore time dependent. However, observing the dynamics of the mitochondrial network dynamics in isolation, without context and reference to the total number of mitochondrial structures, may not be sufficient to describe the overall changes brought about following drug intervention. For this reason, we made further use of MEL to observe the number of mitochondrial structures and the average volume as a means of providing insights into the extent to which the network changes dynamically as well as the overall inter-connectiveness of the mitochondrial network in response to treatment. The average mitochondrial volume of cells treated with HM [0.76 ± 0.04 µm^3^ (*p* < 0.05)], HM + CCCP [0.72 ± 0.04 µm^3^ (*p* < 0.05)] and Baf [0.72 ± 0.05 µm^3^ (*p* < 0.05)] was significantly increased compared to the CCCP treated group (Figure 1C). Baf treatment [58.9 ± 4.9 (*p* < 0.05)] had a similar effect on the count, as was seen in fission and fusion events, resulting in a significant decrease of mitochondrial structures compared to Con [104.5 ± 7.3 (*p* < 0.05)] (Figure 1B). Both LM [87.1 ± 4.9 (*p* < 0.05)] and HM [92.2 ± 12.7 (*p* < 0.05)] counts were significantly higher than Baf but unchanged compared to Con. The addition of CCCP caused no significant change to the mitochondrial structure count compared to that of Con (Figure 1B). In fact, it was expected that there would be an increase in the mitochondrial structures due to the ability of CCCP to induce fission and, subsequently, mitophagy [36,44]. Despite this, we did see a significant decrease in the average mitochondrial volume compared to Con [0.76 ± 0.07 µm^3^ (*p* < 0.05)] after CCCP treatment (Figure 1C). However, mitochondrial structures after LM + CCCP [114.2 ± 13.8 (*p* < 0.05)] were shown to be significantly increased compared to LM and Baf + CCCP [80.9 ± 7.1 (*p* < 0.05)]. This demonstrates that LM maintains average mitochondrial volume but not mitochondrial structures upon CCCP-induced damage, while HM maintains mitochondrial volume similar to control levels, given the presence of CCCP injury.

### 3.3. Autophagy Assessment: Autophagosome, Lysosome and Autolysosome Count and Volume Changes

**Figure 2:**
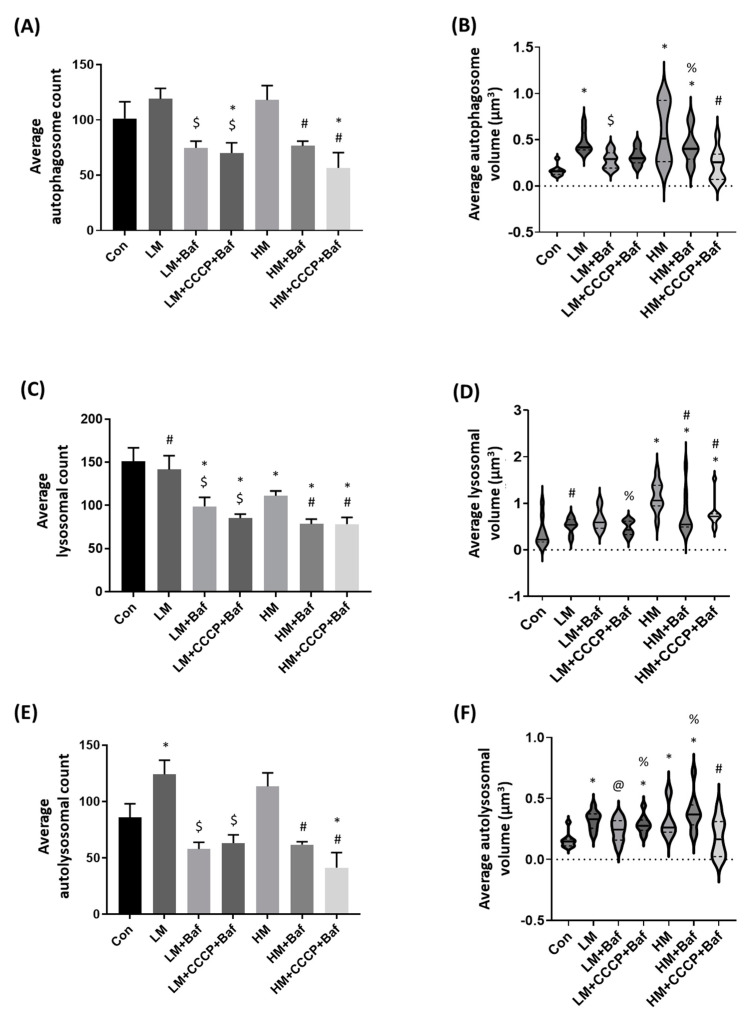

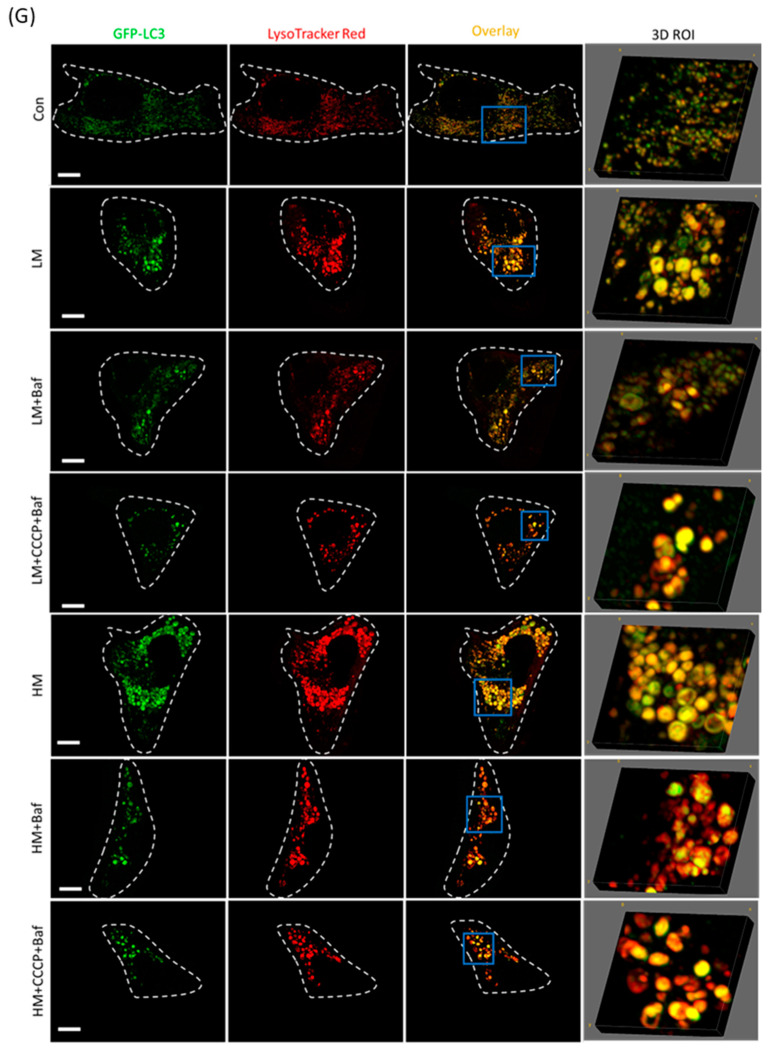
Memantine induces changes to autophagy vesicle counts and sizes. Cells were assessed for their autophagosomal, lysosomal and autolysosomal count changes (**A**,**C**,**E**) as well as for their volume changes in µm^3^ (**B**,**D**,**E**,**F**). Representative MIP micrographs of GFP-LC3 expressing GT1-7 cells stained with LysoTracker Red only (**G**). *n* = 9 cells per treatment group. Results are expressed as mean ± SEM. Scale bar = 10 µm. * *p* < 0.05 vs. Con, # *p* < 0.05 vs. HM, @ *p* < 0.05 vs. HM + B, % *p* < 0.05 vs. HM + C + B and $ *p* < 0.05 vs. LM.

Autophagosome flux is defined as the rate of protein degradation through the entire autophagy pathway, the rate at which autophagosomes are synthesized and subsequently degraded by lysosomes [10]. This is measured by quantifying the change of memantine on autophagosome flux; saturating concentrations of bafilomycin (Baf) were used to deacidify lysosomes [45]. We made use of immunoblotting techniques to probe for the changes of memantine treatment on overall LC3-II and sequestosome/p62 protein abundance. These results were confounding (Figure S1); however, they were less sensitive compared to imaging-based vesicle analysis [46]. Hence, for the purpose of this study, we turned to a single cell analysis approach. CCCP in combination with Baf was employed to dissect the extent of mitophagy induction [43] and to investigate the role of mitochondrial injury on autophagosome flux. Memantine at either concentration did not cause changes to the autophagosomal count. However, both LM [0.47 ± 0.04 µm^3^ (*p* < 0.05)] and HM [0.57 ± 0.11 µm^3^ (*p* < 0.05)] caused a significant increase to the autophagosomal volume against Con, demonstrating a smaller pool size that consists of larger autophagosomes (Figure 2A,B,G). Interestingly, following LM + Baf [74.7 ± 6.1 (*p* < 0.05)] and HM + Baf [76.6 ± 4.3 (*p* < 0.05)], the autophagosome count was seen to decrease against their treatment-only counterparts, whereas the addition of LM + CCCP + Baf [70.1 ± 9.2 (*p* < 0.05)] and HM + CCCP + Baf [56.6 ± 13.9 (*p* < 0.05)] caused a significant decrease in counts against both Con as well as the treatment-only counterparts.

Lysosomes are acidic vesicles that receive autophagosomes and their cargo to form autolysosomes and thereby complete the degradation step in the autophagy process. LM caused no changes to either lysosomal count or volume against Con, whereas HM caused a significant increase to the lysosomal volume, but decreased the count compared to Con. Following HM + Baf [0.78 ± 0.15 µm^3^ (*p* < 0.05] and HM + CCCP + Baf [0.76 ± 0.10 µm^3^ (*p* < 0.05)], the lysosomal volume was shown to remain increased compared to Con, but lower than HM only. This increase in volume was accompanied by a decrease in lysosomal puncta count in both groups, demonstrating an increase in puncta volume in spite of injury following HM. LM [141.6 ± 16.0 (*p* < 0.05)] treatment neither affected lysosomal count nor average lysosomal volume. Furthermore, we noted a decrease in lysosomal count following LM + Baf [98.9 ± 10.5 (*p* < 0.05)] and LM + CCCP + Baf [85.2 ± 4.7 (*p* < 0.05)] compared to those observed in the Con and LM, with no changes to the lysosomal volume.

Autolysosomes treated with both LM [0.32 ± 0.25 µm^3^ (*p* < 0.05)] and HM [0.32 ± 0.05 µm^3^ (*p* < 0.05)] were shown to increase in volume; however, only the AL count of LM [124.0 ± 12.6 (*p* < 0.05)] treated cells were increased against Con (Figure 2E,F). Likewise, a decrease in AL count was detected following treatment with LM + Baf [57.9 ± 6.0 (*p* < 0.05)], LM + CCCP + Baf [63.0 ± 7.4 (*p* < 0.05)], HM + Baf [61.7 ± 2.7 (*p* < 0.05)] and HM + CCCP + Baf [41.3 ± 13.3 (*p* < 0.05)] against their respective, memantine only treated counterparts. AL volume of the LM [0.32 ± 0.03 µm^3^ (*p* < 0.05)] and LM + CCCP + Baf [0.29 ± 0.03 µm^3^ (*p* < 0.05)] treated cells was higher than that of Con cells. AL volume of HM + Baf [0.39 ± 0.05 µm^3^ (*p* < 0.05)] treated cells was increased above those observed in Con cells [0.16 ± 0.02 µm^3^ (*p* < 0.05)], whereas AL volume in HM + CCCP + Baf [0.18 ± 0.05 µm^3^ (*p* < 0.05)] treated cells was smaller than in HM treated cells. HM treated cells presented with an autolysosomal volume [0.32 ± 0.05 µm^3^ (*p* < 0.05)] that was higher than what was seen in the Con group.

### 3.4. Mitophagy Detection

Mitophagy is the degradation of mitochondria through the autophagy pathway, which can be well detected using single cell analyses coupled to fluorescence microscopy [43]. It is known that one role of mitochondrial fission is to isolate dysfunctional mitochondria from those that remain functional [47,48]. It stands to reason that an increased occurrence of fission events may lead to higher levels of mitophagy, especially following the introduction of a stressor such as CCCP [49]. Here, we carefully dissected the colocalisation between autophagosomes and mitochondria, i.e., automitosomes (AM), lysosomes and mitochondria, i.e., mitolysosomes (ML), as well as colocalisation between autophagosomes, mitochondria and lysosomes, i.e., the automitolysosomes (AML); these three structures will collectively be referred to as the mitophagy puncta.

**Figure 3:**
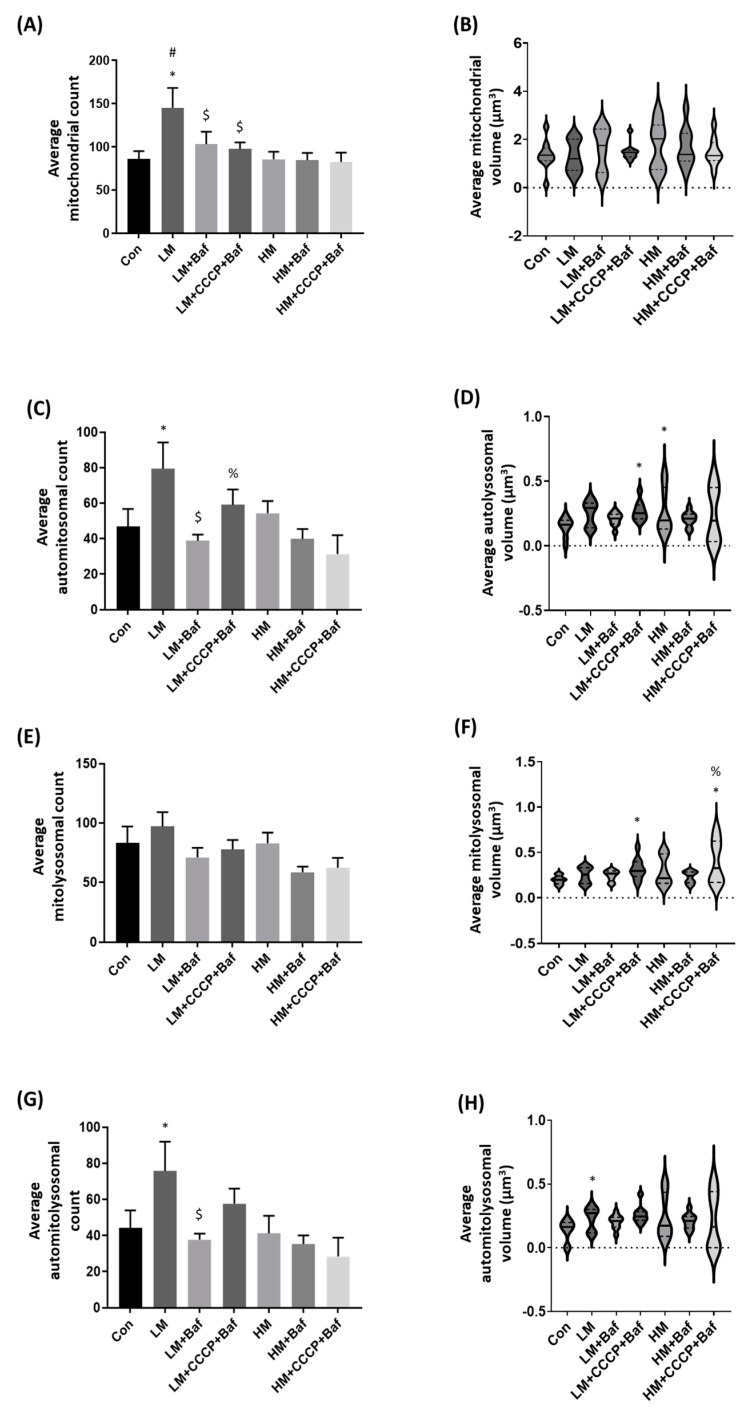

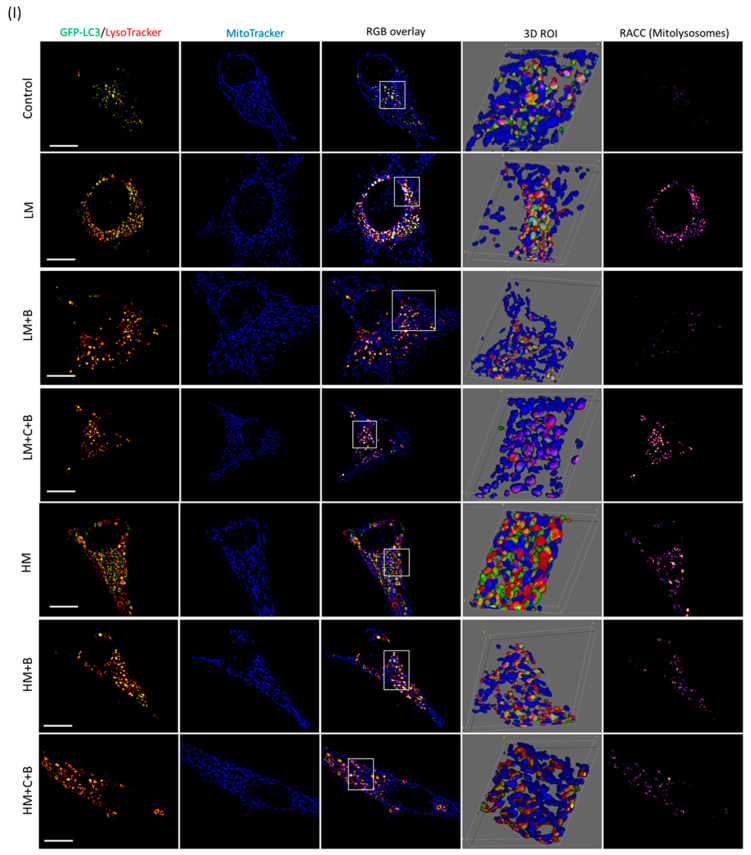
Memantine induces mitophagy in a concentration-dependent manner that differentially impacts vesicle size and puncta count. Mitophagy was assessed using GFP-LC3 transfected GT1-7 cells that were stained with LysoTracker Red and MitoTracker Deep Red, as shown by representative maximum intensity projections (**I**). Mitochondrial structures were counted (**A**) and structure volume was measured (**B**). Subsequently, the colocalisation between structures was analysed and their volumes assessed, including the occurrence of automitosomal formation (**C**,**D**), mitolysosomal formation (**E**,**F**) and automitolysosomal formation (**G**,**H**). *n* = 9 cells per treatment group. Results are expressed as the mean ± SEM. Scale bar = 10 µm. * *p* < 0.05 vs. Con, # *p* < 0.05 vs. HM, % *p* < 0.05 vs. HM + C + B, $ *p* < 0.05 vs. LM.

Following LM treatment, there was a significant increase in AM [51.7 ± 11.8 (*p* < 0.05)] and AML [47.6 ± 12.2 (*p* < 0.05)] counts (Figure 3C,H). ML count following LM treatment [59.9 ± 12.3 (*p* = 0.06)] was increased and neared a significant increase compared to the Con group [21.7 ± 5.1]. Additionally, there was a significant increase in the volume of AM [0.27 ± 0.03 µm^3^ (*p* < 0.05)], ML [0.32 ± 0.04 µm^3^ (*p* < 0.05)] and AML [0.26 ± 0.02 µm^3^ (*p* < 0.05)] following the addition of CCCP and Baf (Figure 3B,D,F). To our surprise, following the addition of HM, no changes were noted in either the count or volume of mitochondrial structures, even in the presence of HM alone [85.6 ± 8.9 and 1.7 ± 0.3 µm^3^], Baf (HM + Baf) [84.6 ± 8.6 and 1.7 ± 0.3 µm^3^] or CCCP and Baf (HM + CCCP + Baf) [82.6 ± 10.8 and 1.5 ± 0.2 µm^3^] (Figure 3A). Although HM did not lead to any significant changes in the numbers of mitophagy puncta, it increased the AM volume [0.27 ± 0.06 µm^3^ (*p* < 0.05)] (Figure 3D) but not the ML volume, whereas HM + CCCP + Baf [0.40 ± 0.08 µm^3^ (*p* < 0.05)] increased the ML volume (Figure 3F).

To gain further confirmation of the extent of mitophagy induction, we implemented the RACC analysis as a means to qualitatively visualize the extent and degree of colocalisation observable between mitochondria and lysosomes [39]. Indeed, we observed a stronger RACC signal when using low concentrations of memantine, increased mitophagy vesicle size and enhanced correlation between the mitochondria and lysosomes (Figure 3). The results suggest that LM engages with mitochondrial quality control whilst HM impacts autophagosome and lysosomal volume.

### 3.5. Correlative Light and Electron Microscopy

**Figure 4:**
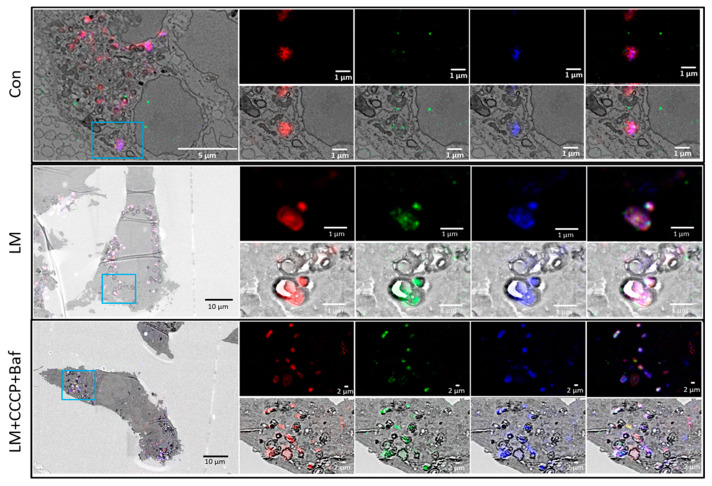

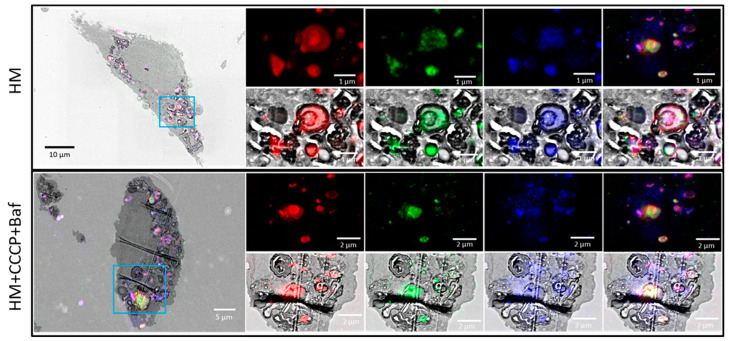
Confirmation of mitophagy induction by memantine using CLEM. CLEM confirmed the increase in mitophagy events that occur following LM treatment as well as the increased number in vacuolar structures following HM treatment. Mitophagy was assessed using GFP-LC3 (green) transfected GT1-7 cells stained with LysoTracker Red (red) and MitoTracker Deep Red (indicated in blue).

While electron microscopy reveals ultrastructural context, the exact molecular identity of the visible structures remains unclear. Although fluorescence probes enable the dissection of specific proteins and organelles of interest, the entire context of the cell is not visualized. To bridge these two microscopy modalities, correlative light and electron microscopy (CLEM) was performed. Our results confirm the notion that LM treated cells present with less vacuolarisation but an indication of increased mitophagy events (Figure 4), confirming the vesicle-based analysis. In stark contrast, HM induces major vacuolarisation, primarily of lysosomal and autolysosomal nature. LM + CCCP + Baf leads to a high abundance of mitophagy events, whereas HM + CCCP + Baf leads to major vacuolarisation, impacting autophagic vacuole size.

## 4. Discussion

Despite major efforts in drug development, no disease modifying treatment currently exists for Alzheimer’s disease. Memantine is one of the major drugs being employed worldwide in the management of the disease, with ongoing global discussions about the recommended dose in the context of disease progression and severity. Autophagy enhancement has been increasingly recognized as a promising avenue to decrease proteotoxicity and to enhance neuronal survival in neurodegeneration [50]. Moreover, its role in mitochondrial quality control has received recent attention [9,47,51]. Here, we assessed whether memantine exhibits a concentration-dependent effect on the autophagy machinery, its pathway intermediates, as well as mitochondrial quality and mitophagy, using an image-based in vitro model approach.

### 4.1. Memantine Causes a Concentration-Dependent Protective Response to Mitochondrial Injury

Previous studies have demonstrated the protective effects of memantine by evaluating mitochondrial-related proteins and functions as a means of demonstrating memantine’s ability to improve cell survival [30,52]. Here, we have made use of the mitochondrial event localizer (MEL) to precisely evaluate the mitochondrial structural changes that take place over time and in three dimensions. Interestingly, fission and fusion event counts changed in a manner similar to mitochondrial structure counts, showing that fission and fusion changes may occur in a manner that is dependent on the total mitochondrial numbers per cell (Figure 1A,B). Treatment with 50 µM (LM) and 100 µM (HM) memantine showed little changes to the fission and fusion events and did not change mitochondrial structure count or the average mitochondrial volume, suggesting that memantine treatment alone does not cause harm to the mitochondrial network.

CCCP was used for its mitochondrial depolarization effects and, to our surprise, did not lead to an increase in fission events on its own [49]. However, we did note a decrease in mitochondrial volume, showing that the network had likely undergone fragmentation prior to observation [44] (Figure 1C). Following co-treatment with CCCP, LM and HM treated cells displayed a concentration-dependent difference in mitochondrial structures and average volume, with LM co-treated with CCCP (LM + CCCP) maintaining volume but leading to a significant increase in the numbers of mitochondria (Figure 1), whilst HM co-treated with CCCP (HM + CCCP) maintaining the number of fission and fusion events, mitochondrial structure counts and the average volume similar to those seen at control. This increase in mitochondrial structures is likely due to the increased numbers of fission events. However, the unchanged mitochondrial volume as well as the increased numbers of fusion events points to a network that remains as active and structured as it was at basal levels, pointing to a mitochondrial network that is well maintained and suited for cell survival.

### 4.2. Memantine Elicits a Differential, Concentration-Dependent Effect on Autophagy Pathway Intermediates

We observe notable differences to the autophagy pathway intermediates, both in their abundance and volume following exposure to memantine at both concentrations compared to control cells (Figure 2). Of particular interest is the increased autolysosomal number and volume following the addition of either memantine concentrations on their own (Figure 2A,B). Past studies assessing autophagy flux only made use of vesicle counts as opposed to observing the size of those counts [38,53,54]. Additionally, memantine has been shown to increase LC3 abundance in a concentration-dependent manner in the absence and presence of a lysosomal inhibitor [28]. However, here, we have shown that memantine elicits a concentration-dependent response to autophagy intermediates in GT1-7 cells with LM increasing both autolysosomal count as well as its volume, whereas HM decreased the pool sizes of autophagosomes but increased the volume of lysosomes and autolysosomes. It stands to reason that larger vesicles would be able to recruit and remove more cytoplasmic cargo, but studies investigating the role of autophagy-related vesicle size and how it impacts autophagy cargo turnover are few [45]. Given that the current definition of autophagy flux takes into account the rate at which autophagosomes are turned over, and not the size of those autophagosomes or how much cargo they carry, it remains unclear to what extent the increase in autophagy intermediate volumes contributes to the overall cargo turnover of the system [55]. The high degree of colocalisation observed between autophagosomes and lysosomes suggests that a large proportion of autophagosomes are indeed in a fused state with lysosomes, which is supported by findings by Jahreiss et al. (2008) [56] and du Toit et al. (2018) [10], supporting the notion that most LC3-positive structures are typically autolysosomes. The fact that autophagy-related vesicles decreased upon Baf treatment (Figure 2) deserves further study. Our Western blot analysis supports a decrease in autophagosome abundance following memantine treatment (Figure S1). It may indeed be the case that memantine affects either lysosomal acidification favourably and, in so doing, delays the onset of effects caused by Baf exposure. Careful dissection of autophagy activity [28] and lysosome acidity status is required. 

### 4.3. Low but Not High Concentrations of Memantine Lead to the Induction of Mitophagy

It is known that mitochondria undergo fission for the purpose of subsequent elimination by mitophagy [30,57], therefore leading to increased mitochondrial structure count. Previous work has shown that memantine enhances mitochondrial protection and may induce mitophagy [28,57]; however, the mitophagy events have not been quantified. The changes observed in mitochondrial network dynamics coupled with the induction of autophagy led us to investigate the possible role of memantine in driving mitophagy. Indeed, it was shown that LM increased the numbers of automitosomes and automitolysosomes (Figure 3C,G), which may account for the increased mitochondrial structure counts (Figure 3A). Additionally, LM led to an increase in automitolysosomal vesicle volume following the application of CCCP and Baf, suggesting the clearance of mitochondrial cargo without increasing the numbers of mitophagy intermediates (Figure 3H). Moreover, we have shown that LM + CCCP treatment increases both the fission and fusion events whilst also maintaining the average mitochondrial volume, whereas HM + CCCP maintains mitochondrial morphometric characteristics similarly to those present at basal levels (Figure 1A–C).

To lend further confidence to an increase and well-defined change in mitophagy, we implemented RACC and CLEM, respectively, and showed a clear increase in correlation in cells treated with LM and LM + CCCP + Baf (Figure 3I). CLEM further confirmed the increased mitophagy events that occurred following LM treatment as well as the increased number in vacuolar structures following HM treatment (Figure 4), confirming the notion that memantine, particularly at a low concentration, robustly induces mitophagy.

## 5. Conclusions

**Figure 5:**
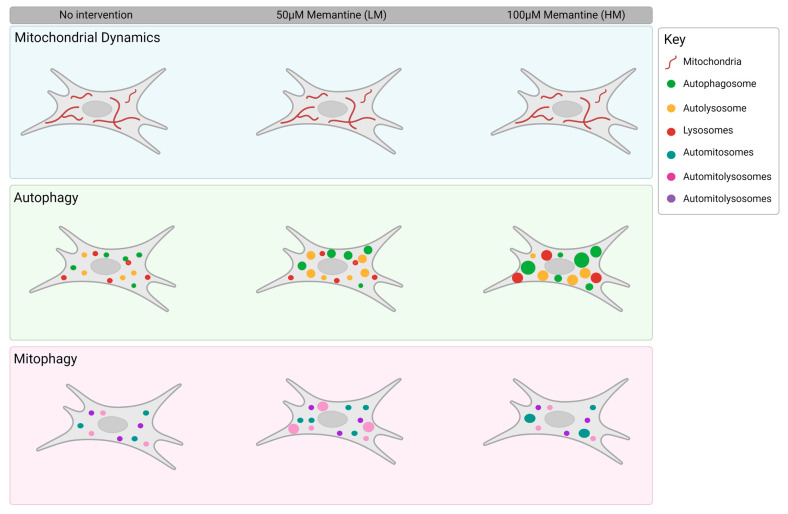
Cartoon depicting the concentration-dependent effects brought about by 100 µM (HM) and 50 µM (LM) memantine treatment. HM treatment resulted in a sharp increase in autophagy vesicle size. Although HM increased automitosomal volume, LM increased the number and volume of automitolysosomes, demonstrating that LM primarily enhances mitophagy, whereas HM leads to an increase in the autophagy compartment. Mitochondrial dynamics remained unchanged following either treatment concentration.

The molecular mechanisms through which memantine impacts the changes in mitochondrial network dynamics appears to be unclear according to the literature. For example, resveratrol, a polyphenol found in the skins of red grapes, has been found to enhance SIRT1α and AMPK [58,59], whereas metformin impacts PGC-1α and AMPK expression levels [60,61], effectively improving the mitochondrial network as well as inducing autophagy. The results presented here suggest that memantine is affecting the autophagy pathway intermediates, as is confirmed in the literature [28,62], albeit with a very finely controlled, concentration-dependent effect on pool size and volume. Specifically, we have demonstrated that memantine acts on autophagy in a concentration-dependent manner which leads to an increase in pathway intermediate vesicle sizes. Additionally, we have shown that while both concentrations of memantine are sufficient to maintain a healthy mitochondrial network, 100 µM of memantine is suitable to increase macroautophagy, whilst 50 µM of memantine is suitable for the induction of mitophagy (Figure 5).

This finding may be beneficial in the clinical context of neurodegenerative diseases, such as Alzheimer’s and Parkinson’s diseases, where dysfunctional mitochondria are an early hallmark of the disease and accumulate within mitochondria whilst still oligomeric [9,63,64]. Given that larger intracellular protein aggregates appear as lysosomal acidification begins to diminish [53,65,66], the lysosomal abundance and volume, in context with autophagy flux and mitochondrial quality control, deserve careful, collective attention.

## 6. Future Outlook

This study took advantage of an in vitro model of neuronal toxicity, allowing for the careful dissection of molecular events associated with autophagy induction, mitophagy and mitochondrial quality control. Further research is required to translate these findings into more complex model systems, including organotypic cultures and the brain in vivo. Currently, such studies are highly limited. Moreover, temporal assessment and longer exposure to memantine will be critical so as to better recreate the clinical environment. Whilst it is known that memantine acts in a neuron-specific manner by engaging with the NMDA receptor, the mechanisms by which memantine would induce autophagy, including the increase of autophagic vacuole size, remain very much unclear. The association of autophagy activity and proteotoxic cargo removal, including aggregated tau protein and Aβ_42_ peptide as well as the crosstalk between mitochondrial mutations and the onset in protein aggregation, as observed in Parkinson’s disease, deserves urgent attention. Given the findings of the present study, additional focus on the molecular targets of memantine is warranted to better unpack the role of memantine in the context of mitochondrial quality control in neuronal injury. Finally, the mitochondrial phenotype may be assessed by using distinct mitochondrial probes simultaneously so as to detect the mitochondrial polarisation status in addition to the respective phenotype. This deserves further study.

## Data Availability

Not applicable.

## Supplementary Materials

The following supporting information can be downloaded at: https://www.mdpi.com/article/10.3390/cells12131726/s1, Figure S1: GFP-LC3 and LysoTracker Red were not successful in indicating autophagy following CCCP and Baf treatment, either separately or in combination. Figure S2: Western blot analysis of relative (A) LC3 and (B) p62 abundance levels of GT1-7 cells treated for 24 h of 100 µM (HME) and 50 µM (LME) of memantine and 48 h with 100 µM (HML) and 50 µM (LML) of memantine. *n* = 3. * *p* < 0.05.

## References

[1] Bjørkøy G., Lamark T., Brech A., Outzen H., Perander M., Øvervatn A., Stenmark H., Johansen T. (2005). p62/SQSTM1 forms protein aggregates degraded by autophagy and has a protective effect on huntingtin-induced cell death. J. Cell Biol..

[2] Kirkin V., Lamark T., Sou Y.-S., Bjørkøy G., Nunn J.L., Bruun J.-A., Shvets E., McEwan D.G., Clausen T.H., Wild P. (2009). A Role for NBR1 in Autophagosomal Degradation of Ubiquitinated Substrates. Mol. Cell.

[3] Serrano-Pozo A., Frosch M.P., Masliah E., Hyman B.T. (2011). Neuropathological Alterations in Alzheimer Disease. Cold Spring Harb. Perspect. Med..

[4] Sharoar G., Hu X., Ma X.-M., Zhu X., Yan R. (2019). Sequential formation of different layers of dystrophic neurites in Alzheimer’s brains. Mol. Psychiatry.

[5] Bence N.F., Sampat R.M., Kopito R.R. (2001). Impairment of the Ubiquitin-Proteasome System by Protein Aggregation. Science.

[6] McNaught K.S., Bjorklund L.M., Belizaire R., Isacson O., Jenner P., Olanow C.W. (2002). Proteasome inhibition causes nigral degeneration with inclusion bodies in rats. Neuroreport.

[7] Rideout H.J., Lang-Rollin I., Stefanis L. (2004). Involvement of macroautophagy in the dissolution of neuronal inclusions. Int. J. Biochem. Cell Biol..

[8] Affaticati P., Mignen O., Jambou F., Potier M.-C., Klingel-Schmitt I., Degrouard J., Peineau S., Gouadon E., Collingridge G.L., Liblau R. (2011). Sustained calcium signalling and caspase-3 activation involve NMDA receptors in thymocytes in contact with dendritic cells. Cell Death Differ..

[9] Cha M.-Y., Han S.-H., Son S.M., Hong H.-S., Choi Y.-J., Byun J., Mook-Jung I. (2012). Mitochondria-Specific Accumulation of Amyloid β Induces Mitochondrial Dysfunction Leading to Apoptotic Cell Death. PLoS ONE.

[10] du Toit A., Hofmeyr J.-H.S., Gniadek T.J., Loos B. (2018). Measuring autophagosome flux. Autophagy.

[11] Loos B., Du Toit A., Hofmeyr J.-H.S. (2014). Defining and measuring autophagosome flux—Concept and reality. Autophagy.

[12] Kim J., Kundu M., Viollet B., Guan K.-L. (2011). AMPK and mTOR regulate autophagy through direct phosphorylation of Ulk1. Nat. Cell Biol..

[13] Pineda-Ramírez N., Alquisiras-Burgos I., Ortiz-Plata A., Ruiz-Tachiquín M.-E., Espinoza-Rojo M., Aguilera P. (2020). Resveratrol Activates Neuronal Autophagy through AMPK in the Ischemic Brain. Mol. Neurobiol..

[14] Arribat Y., Broskey N.T., Greggio C., Boutant M., Alonso S.C., Kulkarni S.S., Lagarrigue S., Carnero E.A., Besson C., Cantó C. (2019). Distinct patterns of skeletal muscle mitochondria fusion, fission and mitophagy upon duration of exercise training. Acta Physiol..

[15] Bernhardt D., Müller M., Reichert A.S., Osiewacz H.D. (2015). Simultaneous impairment of mitochondrial fission and fusion reduces mitophagy and shortens replicative lifespan. Sci. Rep..

[16] Park J., Lee J., Choi C. (2011). Mitochondrial Network Determines Intracellular ROS Dynamics and Sensitivity to Oxidative Stress through Switching Inter-Mitochondrial Messengers. PLoS ONE.

[17] Parone P.A., Da Cruz S., Tondera D., Mattenberger Y., James D.I., Maechler P., Barja F., Martinou J.-C. (2008). Preventing Mitochondrial Fission Impairs Mitochondrial Function and Leads to Loss of Mitochondrial DNA. PLoS ONE.

[18] Mourier A., Motori E., Brandt T., Lagouge M., Atanassov I., Galinier A., Rappl G., Brodesser S., Hultenby K., Dieterich C. (2015). Mitofusin 2 is required to maintain mitochondrial coenzyme Q levels. J. Cell Biol..

[19] Chung S.Y., Kishinevsky S., Mazzulli J.R., Graziotto J., Mrejeru A., Mosharov E.V., Puspita L., Valiulahi P., Sulzer D., Milner T.A. (2016). Parkin and PINK1 Patient iPSC-Derived Midbrain Dopamine Neurons Exhibit Mitochondrial Dysfunction and α-Synuclein Accumulation. Stem Cell Rep..

[20] Tammineni P., Jeong Y.Y., Feng T., Aikal D., Cai Q. (2017). Impaired axonal retrograde trafficking of the retromer complex augments lysosomal deficits in Alzheimer’s disease neurons. Hum. Mol. Genet..

[21] Martinez-Vicente M., Talloczy Z., Wong E., Tang G., Koga H., Kaushik S., De Vries R., Arias E., Harris S., Sulzer D. (2010). Cargo recognition failure is responsible for inefficient autophagy in Huntington’s disease. Nat. Neur..

[22] Kumar R., Amruthanjali T., Singothu S., Singh S.B., Bhandari V. (2022). Uncoupling proteins as a therapeutic target for the development of new era drugs against neurodegenerative disorder. Biomed. Pharmacother..

[23] Monteiro-Cardoso V.F., Oliveira M.M., Melo T., Domingues M.R., Moreira P.I., Ferreiro E., Peixoto F., Videira R.A. (2014). Cardiolipin Profile Changes are Associated to the Early Synaptic Mitochondrial Dysfunction in Alzheimer’s Disease. J. Alzheimer’s Dis..

[24] Danysz W., Parsons C.G., Möbius H.-J., Stöffler A., Quack G. (2000). Neuroprotective and symptomatological action of memantine relevant for alzheimer’s disease—A unified glutamatergic hypothesis on the mechanism of action. Neurotox. Res..

[25] Shankar G.M., Bloodgood B.L., Townsend M., Walsh D.M., Selkoe D.J., Sabatini B.L. (2007). Natural Oligomers of the Alzheimer Amyloid-β Protein Induce Reversible Synapse Loss by Modulating an NMDA-Type Glutamate Receptor-Dependent Signaling Pathway. J. Neurosci..

[26] Grossberg G.T., Manes F., Allegri R.F., Gutiérrez-Robledo L.M., Gloger S., Xie L., Jia X.D., Pejović V., Miller M.L., Perhach J.L. (2013). The Safety, Tolerability, and Efficacy of Once-Daily Memantine (28 mg): A Multinational, Randomized, Double-Blind, Placebo-Controlled Trial in Patients with Moderate-to-Severe Alzheimer’s Disease Taking Cholinesterase Inhibitors. CNS Drugs.

[27] Skeberdis V.A., Chevaleyre V., Lau C.G., Goldberg J.H., Pettit D.L., Suadicani S.O., Lin Y., Bennett M.V.L., Yuste R., Castillo P.E. (2006). Protein kinase A regulates calcium permeability of NMDA receptors. Nat. Neurosci..

[28] Hirano K., Fujimaki M., Sasazawa Y., Yamaguchi A., Ishikawa K.-I., Miyamoto K., Souma S., Furuya N., Imamichi Y., Yamada D. (2019). Neuroprotective effects of memantine via enhancement of autophagy. Biochem. Biophys. Res. Commun..

[29] Song G., Li Y., Lin L., Cao Y. (2015). Anti-autophagic and anti-apoptotic effects of memantine in a SH-SY5Y cell model of Alzheimer’s disease via mammalian target of rapamycin-dependent and -independent pathways. Mol. Med. Rep..

[30] Wang Y., Jiang B., Luo W. (2022). Memantine ameliorates oxaliplatin-induced neurotoxicity via mitochondrial protection. Bioengineered.

[31] Ju C., Wong I.C.K., Lau W.C.Y., Man K.K.C., Brauer R., Ma T., Alsharif A., Alwafi H., Lau K.K., Chan E.W. (2021). Global trends in symptomatic medication use against dementia in 66 countries/regions from 2008 to 2018. Eur. J. Neurol..

[32] Hardingham G.E., Bading H. (2010). Synaptic versus extrasynaptic NMDA receptor signalling: Implications for neurodegenerative disorders. Nat. Rev. Neurosci..

[33] Brewer L., Bennett K., McGreevy C., Williams D. (2013). A population-based study of dosing and persistence with anti-dementia medications. Eur. J. Clin. Pharmacol..

[34] Dolder C., Nelson M., McKinsey J. (2009). Memantine Dosing in Patients with Dementia. Am. J. Geriatr. Psychiatry.

[35] Elias A.M., Pepin M.J., Brown J.N. (2019). Adjunctive memantine for opioid use disorder treatment: A systematic review. J. Subst. Abus. Treat..

[36] Lang A., Anand R., Altinoluk-Hambüchen S., Ezzahoini H., Stefanski A., Iram A., Bergmann L., Urbach J., Böhler P., Hänsel J. (2017). SIRT4 interacts with OPA1 and regulates mitochondrial quality control and mitophagy. Aging.

[37] Theart R.P., Kriel J., Du Toit A., Loos B., Niesler T.R. (2020). Mitochondrial event localiser (MEL) to quantitativelydescribe fission, fusion and depolarisation in the three-dimensional space. PLoS ONE.

[38] de Wet S., Du Toit A., Loos B. (2021). Spermidine and Rapamycin Reveal Distinct Autophagy Flux Response and Cargo Receptor Clearance Profile. Cells.

[39] Theart R., Loos B., Niesler T.R. (2019). Regression adjusted colocalisation colour mapping (RACC): A novel biological visual analysis method for qualitative colocalisation analysis of 3D fluorescence micrographs. PLoS ONE.

[40] Peddie C.J., Blight K., Wilson E., Melia C., Marrison J., Carzaniga R., Domart M.-C., O’Toole P., Larijani B., Collinson L.M. (2014). Correlative and integrated light and electron microscopy of in-resin GFP fluorescence, used to localise diacylglycerol in mammalian cells. Ultramicroscopy.

[41] Kriel J., Lumkwana D., Joubert L.-M., Jones M.L., Peddie C.J., Collinson L., Loos B., Engelbrecht L. (2022). Imaging and Quantifying Neuronal Autophagy.

[42] Paul-Gilloteaux P., Heiligenstein X., Belle M., Domart M.-C., Larijani B., Collinson L., Raposo G., Salamero J. (2017). eC-CLEM: Flexible multidimensional registration software for correlative microscopies. Nature Methods..

[43] Dolman N.J., Chambers K.M., Mandavilli B., Batchelor R.H., Janes M.S. (2013). Tools and Techniques to measure mitophagy using fluorescence microscopy. Autophagy.

[44] Li G.-B., Zhang H.-W., Fu R.-Q., Hu X.-Y., Liu L., Li Y.-N., Liu Y.-X., Liu X., Hu J.-J., Deng Q. (2018). Mitochondrial fission and mitophagy depend on cofilin-mediated actin depolymerization activity at the mitochondrial fission site. Oncogene.

[45] Boland B., Kumar A., Lee S., Platt F.M., Wegiel J., Yu W.H., Nixon R.A. (2008). Autophagy Induction and Autophagosome Clearance in Neurons: Relationship to Autophagic Pathology in Alzheimer’s Disease. J. Neurosci..

[46] Lumkwana D., Peddie C., Kriel J., Michie L.L., Heathcote N., Collinson L., Kinnear C., Loos B. (2022). Investigating the Role of Spermidine in a Model System of Alzheimer’s Disease Using Correlative Microscopy and Super-resolution Techniques. Front. Cell Dev. Biol..

[47] Frank M., Duvezin-Caubet S., Koob S., Occhipinti A., Jagasia R., Petcherski A., Ruonala M.O., Priault M., Salin B., Reichert A.S. (2012). Mitophagy is triggered by mild oxidative stress in a mitochondrial fission dependent manner. Biochim. Biophys. Acta (BBA) Mol. Cell Res..

[48] Gomes L.C., Scorrano L. (2008). High levels of Fis1, a pro-fission mitochondrial protein, trigger autophagy. Biochim. Biophys. Acta Bioenerg..

[49] Ding W.-X., Ni H.-M., Li M., Liao Y., Chen X., Stolz D.B., Dorn G.W., Yin X.-M. (2010). Nix Is Critical to Two Distinct Phases of Mitophagy, Reactive Oxygen Species-mediated Autophagy Induction and Parkin-Ubiquitin-p62-mediated Mitochondrial Priming. J. Biol. Chem..

[50] Koentjoro B., Park J.-S., Sue C.M. (2017). Nix restores mitophagy and mitochondrial function to protect against PINK1/Parkin-related Parkinson’s disease. Sci. Rep..

[51] Evans C.S., Holzbaur E.L. (2020). Quality Control in Neurons: Mitophagy and Other Selective Autophagy Mechanisms. J. Mol. Biol..

[52] Chen T.-F., Tang M.-C., Chou C.-H., Chiu M.-J., Huang R.-F. (2013). Dose-dependent folic acid and memantine treatments promote synergistic or additive protection against Aβ(25–35) peptide-induced apoptosis in SH-SY5Y cells mediated by mitochondria stress-associated death signals. Food Chem. Toxicol..

[53] Bordi M., Berg M.J., Mohan P.S., Peterhoff C.M., Alldred M.J., Che S., Ginsberg S.D., Nixon R.A. (2016). Autophagy flux in CA1 neurons of Alzheimer hippocampus: Increased induction overburdens failing lysosomes to propel neuritic dystrophy. Autophagy.

[54] Sarkar C., Zhao Z., Aungst S., Sabirzhanov B., Faden A.I., Lipinski M.M. (2014). Impaired autophagy flux is associated with neuronal cell death after traumatic brain injury. Autophagy.

[55] Lumkwana D., du Toit A., Kinnear C., Loos B. (2017). Autophagic flux control in neurodegeneration: Progress and precision targeting—Where do we stand?. Prog. Neurobiol..

[56] Jahreiss L., Menzies F.M., Rubinsztein D.C. (2008). The itinerary of autophagosomes: From peripheral formation to kiss-and-run fusion with lysosomes. Prog. Neurobiol..

[57] Twig G., Elorza A., Molina A.J.A., Mohamed H., Wikstrom J.D., Walzer G., Stiles L., Haigh S.E., Katz S., Las G. (2008). Fission and selective fusion govern mitochondrial segregation and elimination by autophagy. EMBO J..

[58] Park S.-J., Ahmad F., Philp A., Baar K., Williams T., Luo H., Ke H., Rehmann H., Taussig R., Brown A.L. (2012). Resveratrol Ameliorates Aging-Related Metabolic Phenotypes by Inhibiting cAMP Phosphodiesterases. Cell.

[59] Price N.L., Gomes A.P., Ling A.J., Duarte F.V., Martin-Montalvo A., North B.J., Agarwal B., Ye L., Ramadori G., Teodoro J.S. (2012). SIRT1 Is Required for AMPK Activation and the Beneficial Effects of Resveratrol on Mitochondrial Function. Cell Metab..

[60] Izzo A., Nitti M., Mollo N., Paladino S., Procaccini C., Faicchia D., Calì G., Genesio R., Bonfiglio F., Cicatiello R. (2017). Metformin restores the mitochondrial network and reverses mitochondrial dysfunction in Down syndrome cells. Hum. Mol. Genet..

[61] Shi W.-Y., Xiao D., Wang L., Dong L.-H., Yan Z.-X., Shen Z.-X., Chen S.-J., Chen Y., Zhao W.-L. (2012). Therapeutic metformin/AMPK activation blocked lymphoma cell growth via inhibition of mTOR pathway and induction of autophagy. Cell Death Dis..

[62] Sestito S., Daniele S., Pietrobono D., Citi V., Bellusci L., Chiellini G., Calderone V., Martini C., Rapposelli S. (2019). Memantine prodrug as a new agent for Alzheimer’s Disease. Sci. Rep..

[63] Burman J.L., Pickles S., Wang C., Sekine S., Vargas J.N.S., Zhang Z., Youle A.M., Nezich C.L., Wu X., Hammer J.A. (2017). Mitochondrial fission facilitates the selective mitophagy of protein aggregates. J. Cell Biol..

[64] Manczak M., Anekonda T.S., Henson E., Park B.S., Quinn J., Reddy P.H. (2006). Mitochondria are a direct site of Aβ accumulation in Alzheimer’s disease neurons: Implications for free radical generation and oxidative damage in disease progression. Hum. Mol. Genet..

[65] Lee S., Sato Y., Nixon R.A. (2011). Lysosomal Proteolysis Inhibition Selectively Disrupts Axonal Transport of Degradative Organelles and Causes an Alzheimer’s-Like Axonal Dystrophy. J. Neurosci..

[66] Lee J.-H., Yang D.-S., Goulbourne C.N., Im E., Stavrides P., Pensalfini A., Chan H., Bouchet-Marquis C., Bleiwas C., Berg M.J. (2022). Faulty autolysosome acidification in Alzheimer’s disease mouse models induces autophagic build-up of Aβ in neurons, yielding senile plaques. Nat. Neurosci..

