# Supplementary material for: The Highs and Lows of Memantine—An Autophagy and Mitophagy Inducing Agent That Protects Mitochondria
Source: Cells. 2023 Jun 27;12(13):1726. doi: 10.3390/cells12131726 (PMC10340721; doi:10.3390/cells12131726)
Supplement: Supplementary file 1 [file cells-12-01726-s001.zip › cells-2366553-supplementary.pptx]

## Slide 1
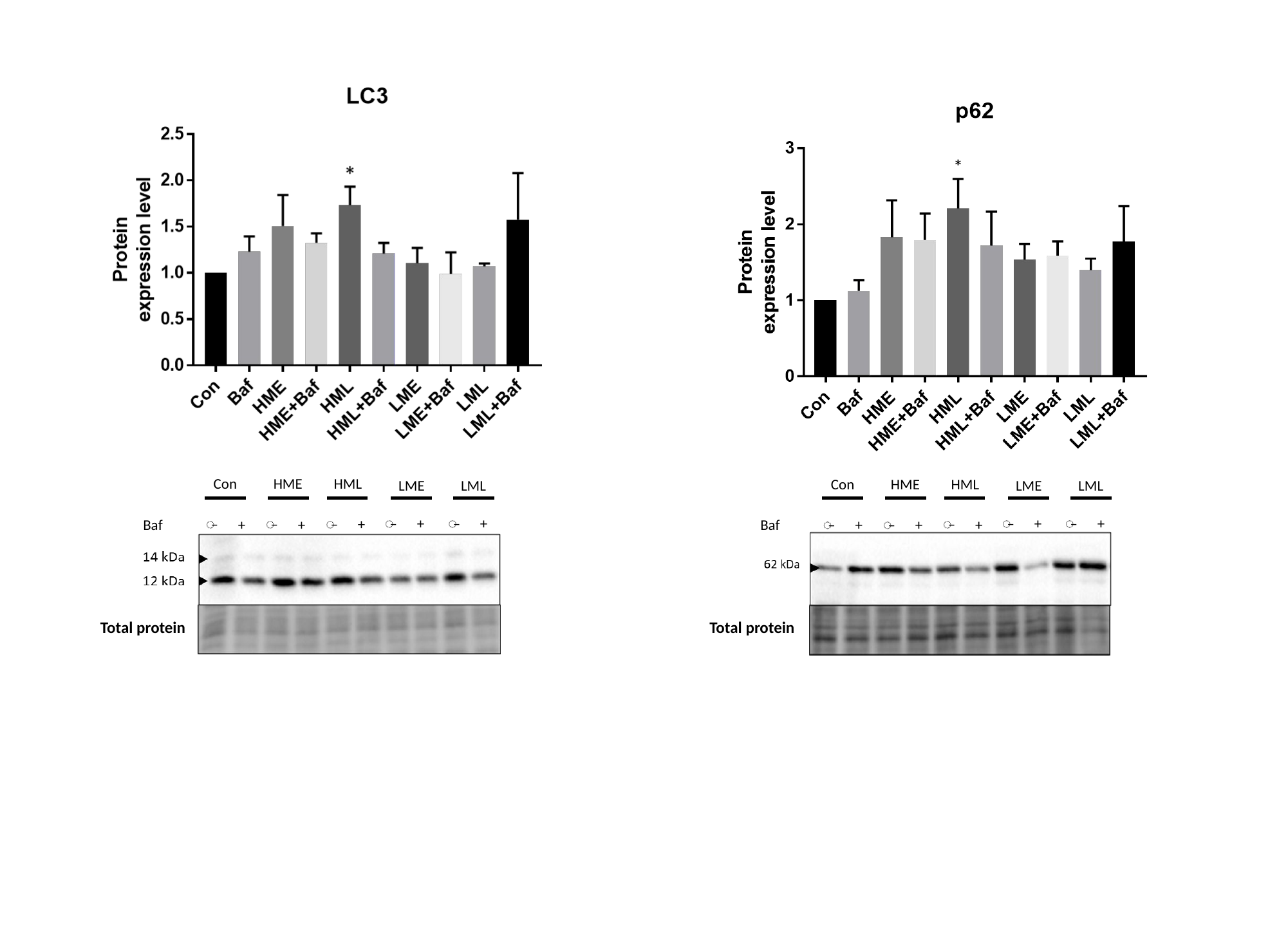

Con
HME
HML
Con
HME
HML
LME
LML
LME
LML
̶
̶
̶
+
̶
+
+
+
̶
̶
̶
̶
+
̶
̶
+
Baf
+
+
Baf
+
+
Total protein
Total protein

## Slide 2
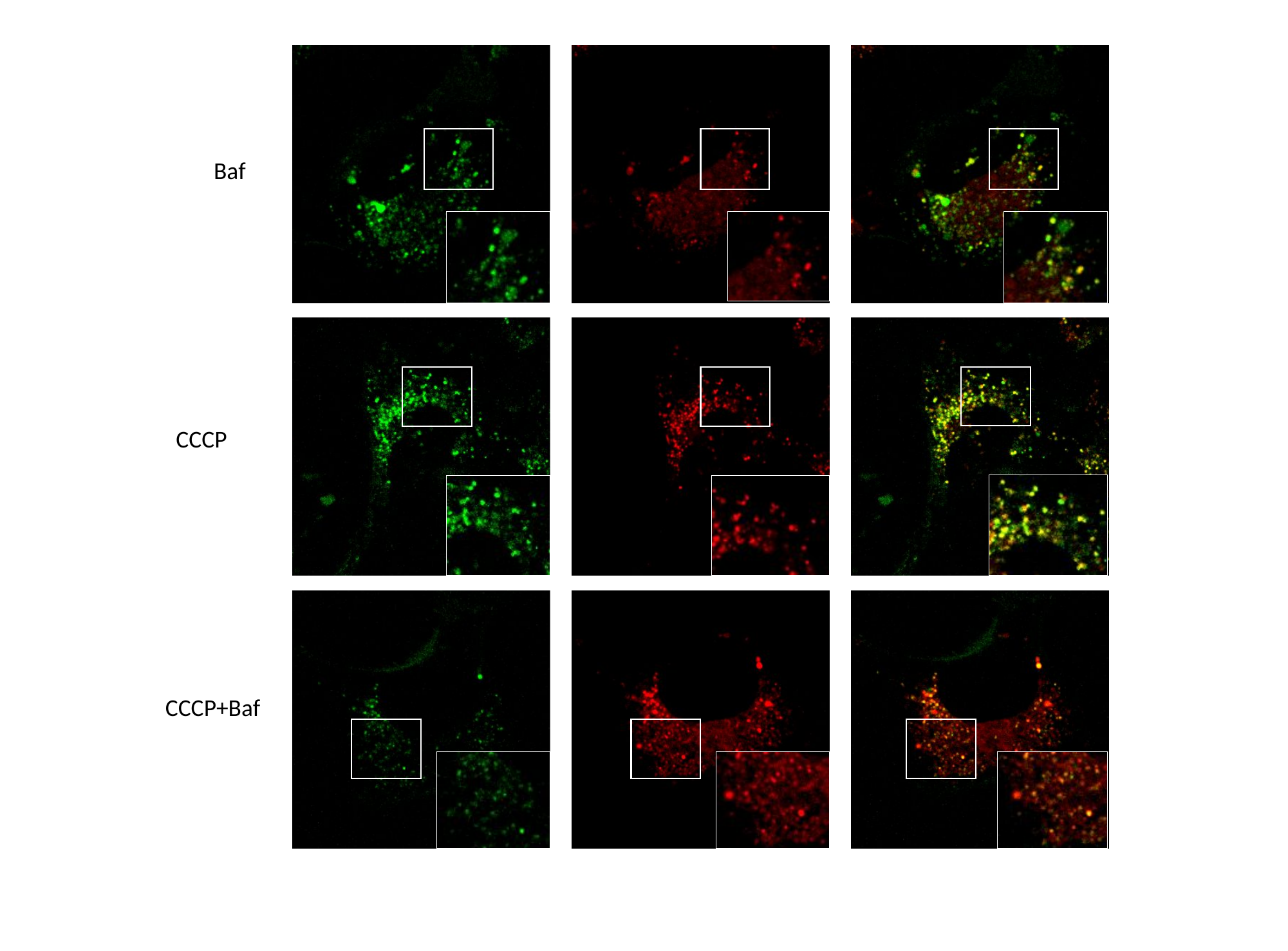

Baf
CCCP
CCCP+Baf
